# Remodeling of the Fibrillation Pathway of α‐Synuclein by Interaction with Antimicrobial Peptide LL‐III

**DOI:** 10.1002/chem.202101592

**Published:** 2021-07-22

**Authors:** Rosario Oliva, Sanjib K. Mukherjee, Lena Ostermeier, Lilli A. Pazurek, Simon Kriegler, Verian Bader, Daniel Prumbaum, Stefan Raunser, Konstanze F. Winklhofer, Jörg Tatzelt, Roland Winter

**Affiliations:** ^1^ Physical Chemistry I – Biophysical Chemistry Faculty of Chemistry and Chemical Biology TU Dortmund University Otto-Hahn-Strasse 4a 44227 Dortmund Germany; ^2^ Department Biochemistry of Neurodegenerative Diseases Institute of Biochemistry and Pathobiochemistry Ruhr University Bochum Germany; ^3^ Department Molecular Cell Biology Institute of Biochemistry and Pathobiochemistry Ruhr University Bochum Germany; ^4^ Department of Structural Biochemistry Max Planck Institute of Molecular Physiology Otto-Hahn-Strasse 11 44227 Dortmund Germany

**Keywords:** α-synuclein, antimicrobial peptide, Lewy bodies, liquid-liquid phase separation, LL-III, protein condensate

## Abstract

Liquid‐liquid phase separation (LLPS) has emerged as a key mechanism for intracellular organization, and many recent studies have provided important insights into the role of LLPS in cell biology. There is also evidence that LLPS is associated with a variety of medical conditions, including neurodegenerative disorders. Pathological aggregation of α‐synuclein, which is causally linked to Parkinson's disease, can proceed via droplet condensation, which then gradually transitions to the amyloid state. We show that the antimicrobial peptide LL‐III is able to interact with both monomers and condensates of α‐synuclein, leading to stabilization of the droplet and preventing conversion to the fibrillar state. The anti‐aggregation activity of LL‐III was also confirmed in a cellular model. We anticipate that studying the interaction of antimicrobial‐type peptides with liquid condensates such as α‐synuclein will contribute to the understanding of disease mechanisms (that arise in such condensates) and may also open up exciting new avenues for intervention.

## Graphical abstract

Avoiding fibrils: The cationic antimicrobial peptide LL‐III stabilizes droplet condensates formed by α‐synuclein, thereby impeding their transformation to the fibrillar amyloid state.

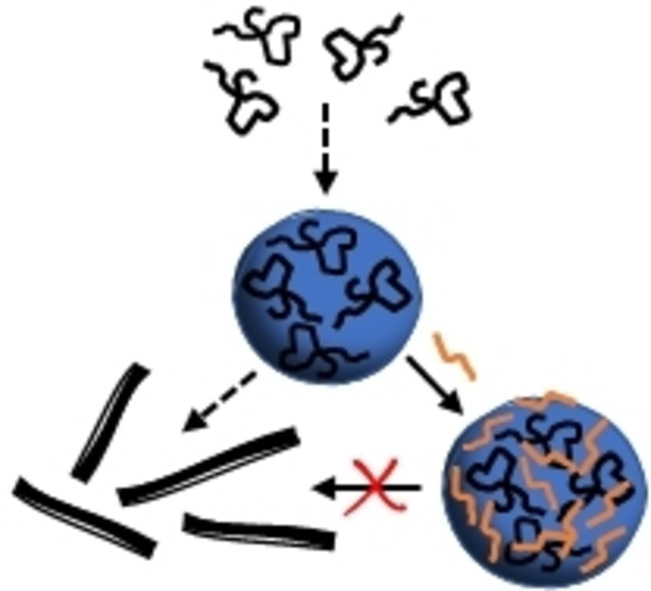

## Introduction

α‐Synuclein (α‐Syn) is a small intrinsically disordered presynaptic protein which regulates neurotransmitter vesicle cycling. Misfolding and aggregation of α‐Syn is associated with Parkinson's disease (PD), reflected by the presence of Lewy bodies as a pathological hallmark of PD and other synucleinopathies, such as multiple system atrophy and dementia with Lewy bodies.[^1^, ^2^, ^3^, ^4^] Human α‐Syn protein has 140 amino acid residues and consists of three distinct regions, which include an amphipathic N‐terminal domain (residues 1–60), a central hydrophobic region, the non‐Aβ component (NAC) region (residues 61–95), and a highly negatively charged proline‐rich C‐terminal domain (residues 96–140). The N‐terminal and hydrophobic regions of α‐Syn are known to adopt an α‐helical conformation when bound to negatively charged vesicles.^[5]^

Though there is a large body of evidence showing that α‐Syn can aggregate into amyloid fibrils, the relationship between α‐Syn′s self‐assembly and Lewy body formation remains still largely unclear. Recently, it has been shown that α‐Syn is also able to undergo liquid‐liquid phase separation (LLPS) by forming a liquid droplet state, which converts with time into an amyloid‐rich hydrogel with Lewy body‐like properties.[^6^, ^7^, ^8^, ^9^] Generally, under healthy conditions, the condensation of proteins into a dense liquid droplet state through LLPS is reversible and exploited in many ways to regulate cellular functions in a spatio‐temporal manner, including RNA metabolism, ribosome biogenesis, DNA damage response, and signal transduction.[^10^, ^11^, ^12^, ^13^] Upon dysregulation, however, liquid droplets can mature into gel‐like deposits, which irreversibly sequester essential cellular components and lead to pathological processes. Such mechanism has been shown to drive the self‐assembly of various other disease‐associated proteins on the pathway to the formation of solid aggregates. This phenomenon has, for example, been observed for FUS and TDP‐43 in amyotrophic lateral sclerosis and frontotemporal dementia, for tau in Alzheimer's disease, for IAPP in diabetes mellitus type II, and for PrP in prion diseases.[^14^, ^15^, ^16^, ^17^, ^18^, ^19^, ^20^, ^21^, ^22^, ^23^, ^24^]

The underlying mechanisms of the disease initiation and progression are still poorly understood. This gap in knowledge of the disease aetiology makes it difficult to pick a target for drug discovery and is therefore partly responsible for the current lack of therapies. A promising approach would be to inhibit the conversion from the liquid to the solid (fibrillar) phase. In this work, we explored the effect of an antimicrobial peptide on LLPS and fibril formation of α‐Syn. The interaction of antimicrobial peptides with LLPS phenomena and fibril formation is still *terra incognita* and is hence in the focus of this work.

Antimicrobial peptides (AMPs) are a particular class of disordered proteins. They form an important part of the immune system of all living organisms and are considered as the first line of defense against infections caused by pathogens.[^25^, ^26^, ^27^] Here we used the AMP LL‐III, which has been extracted from the venom of the eusocial bee *Lasioglossum laticeps*, and shows a remarkable activity against several bacterial strains, fungi and cancer cells, coupled with a moderate cytotoxicity which renders this peptide a good candidate for pharmacological applications.^[28]^ The LL‐III is a positively charged (+6 at pH 7.4, also at the lower pH of pH 5.0), unstructured peptide composed of 15 amino acids, half of which are hydrophobic residues and half are polar (with five Lys residues). Interestingly, it was demonstrated that LL‐III is also able to translocate across the plasma membrane of cancer cells, localizing mainly in the nucleolus and in granules that are formed through LLPS of proteins and nucleic acids.^[22]^ Motivated by this observation, we set out to investigate the effect of the LL‐III peptide on the phase separation and fibrillation propensity of α‐Syn employing both various biophysical and cell biological tools.

## Results and Discussion

**Figure 1:**
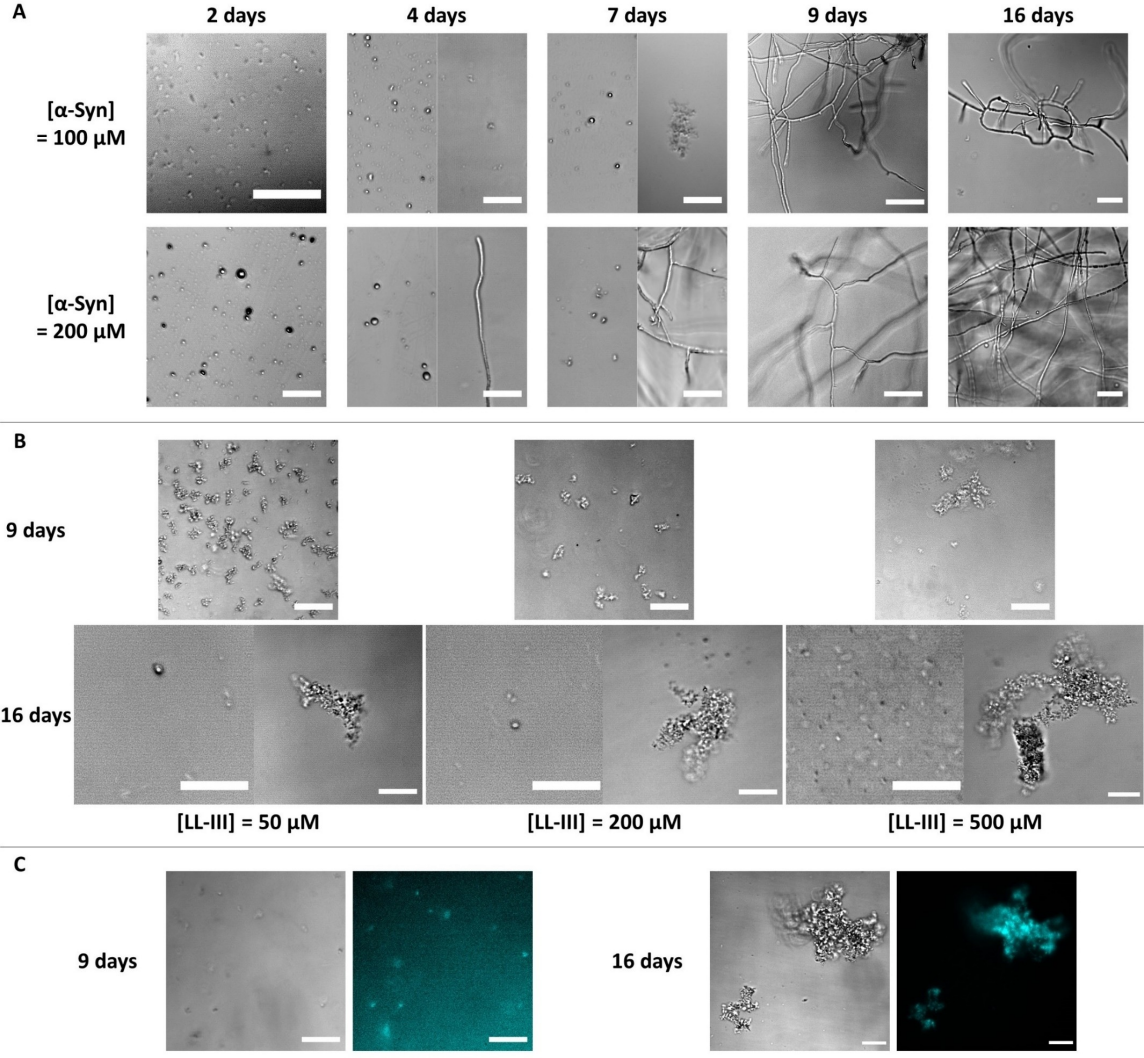
A) Phase‐contrast light microscopy snapshots of 100 μM and 200 μM α‐Syn solutions collected 2, 4, 7, 9 and 16 days after incubation of the monomeric α‐Syn. B) Phase‐contrast light microscopy snapshots of a solution of 100 μM α‐Syn in the presence of the reported concentration of antimicrobial peptide LL‐III. Each sample was prepared by mixing the monomeric form of α‐Syn with LL‐III. The pictures were taken after 9 and 16 days of incubation. For the snapshots after 16 days, at each peptide concentration, two different regions of the cover slip were sampled, showing the presence of discrete droplets as well as droplet clusters in solution. C) Phase‐contrast light and fluorescence microscopy snapshots of a 100 μM α‐Syn solution in the presence of 50 μM LL‐III (total peptide concentration, the concentration of the carboxyfluorescein‐labelled peptide was 1 μM). All the experiments were performed at room temperature (25 °C) in 20 mM sodium acetate buffer, pH 5.0. The scale bar is 30 μm.

Previously, it was shown that α‐Syn at acidic pH is readily able to phase separate, forming sub‐micrometer sized droplets already at 10 μM concentration.^[8]^ At physiological pH, phase separation occurs at much higher concentrations.^[8]^ We first verified that solutions containing 100 μM and 200 μM α‐Syn, at pH 5.0, were able to effectively form droplets. Figure 1A shows phase‐contrast light microscopy images of the two α‐Syn solutions taken 2, 4, 7, 9 and 16 days after sample preparation.

After 2 days of incubation of the monomeric polypeptide at 100 μM α‐Syn, round‐shaped μm‐sized (∼1 μm) droplets of α‐Syn were detected, in agreement with previous observations.^[8]^ After 4 days, slightly bigger droplets were still visible, with an average size of 2 μm. In addition, some small droplet clusters were also present. A similar scenario was still observed after 7 days, however, the droplet clusters were bigger in size (∼4 μm). After 9 days, formation of elongated fibrils was clearly visible and essentially no droplets were detected anymore. Qualitatively, after 16 days no further changes were observed, indicating that the fibrils are stable in time and that, most likely, the process of fibril formation was already completed after ∼9 days. At 200 μM α‐Syn, after 2 days, larger droplets were observed compared to the sample at 100 μM. After 4 days, both droplets and fibrils were present. After 7 days, the number of droplets seemed to be reduced, but more fibrillar structures were visible. Finally, after 9 days only fibrils were observed which remained stable even after 16 days. Thus, as expected, the formation of droplets was faster at higher α‐Syn concentrations. To further characterize fibril formation of α‐Syn, transmission electron microscopy (TEM) experiments were carried out after 9 days of incubation of the monomeric protein (reported in Figure S1). The formation of μm‐sized, elongated fibrils was clearly observed, confirming the tendency of this protein to form elongated fibrillar structures under such solution conditions. Moreover, some droplets were still visible (Figure S1, D), supporting the previous observation that the transition from the monomeric to the fibrillar state occurs through the formation of α‐Syn condensates.^[8]^

To reveal if the short antimicrobial peptide LL‐III is able to affect the fibril formation of α‐Syn, we performed a series of microscopy experiments by using 100 μM of α‐Syn and varying the concentration of LL‐III between 50 μM and 500 μM. The samples were prepared by mixing a solution of LL‐III peptide and α‐Syn in the monomeric state and taking pictures after 9 and 16 days. The results are collected in Figure 1B. Remarkably, we found that when LL‐III was added to the monomeric α‐Syn solution, the protein was no longer able to transform to the fibrillar state. This is to say, LL‐III inhibits formation of the amyloidogenic state of the protein. After 9 days, droplets as well as clusters thereof were visible. The size of the clusters depended on the LL‐III concentration. Higher peptide concentrations promoted the association of more droplets. Further, the microscopy pictures recorded after 16 days showed that the addition of LL‐III fostered formation of the droplet condensate, most likely by largely partitioning inside the droplet phase. This indicates that the LL‐III peptide interacts efficiently with monomeric α‐Syn in the droplet phase, thereby stabilizing the condensate and preventing maturation to the fibrillar state. The microscopy data revealed that large clusters of droplets were finally forming at a later stage of the process (after 16 days) at high droplet concentrations. To confirm partitioning of the LL‐III peptide in the α‐Syn droplets, complementary fluorescence microscopy experiments were performed using a carboxyfluorescein‐labelled LL‐III peptide. Figure 1C shows that most of the LL‐III peptide was localized in the α‐Syn droplets, and the remaining LL‐III was seen in the bulk solution. Qualitatively, similar results were obtained in the presence of 200 μM and 500 μM LL‐III (Figure S2). In agreement with the phase‐contrast microscopy results, formation of LL‐III‐enriched droplet clusters were observed after 16 days.

To verify inhibition of α‐Syn fibril formation upon addition of LL‐III, atomic force microscopy (AFM) experiments were carried out. To this end, a 20 μM solution of α‐Syn in the absence and in the presence of LL‐III was dried on a mica surface and AFM pictures were recorded. Under such conditions, liquid droplets could not be detected. In the absence of LL‐III, short fibril‐like structures were observed (Figure S3). In the presence of 200 μM LL‐III, some fibrils of smaller size were still visible after drying. At 500 μM LL‐III, fibrillar structures could no longer be detected, confirming the phase‐contrast light microscopy images.

**Figure 2:**
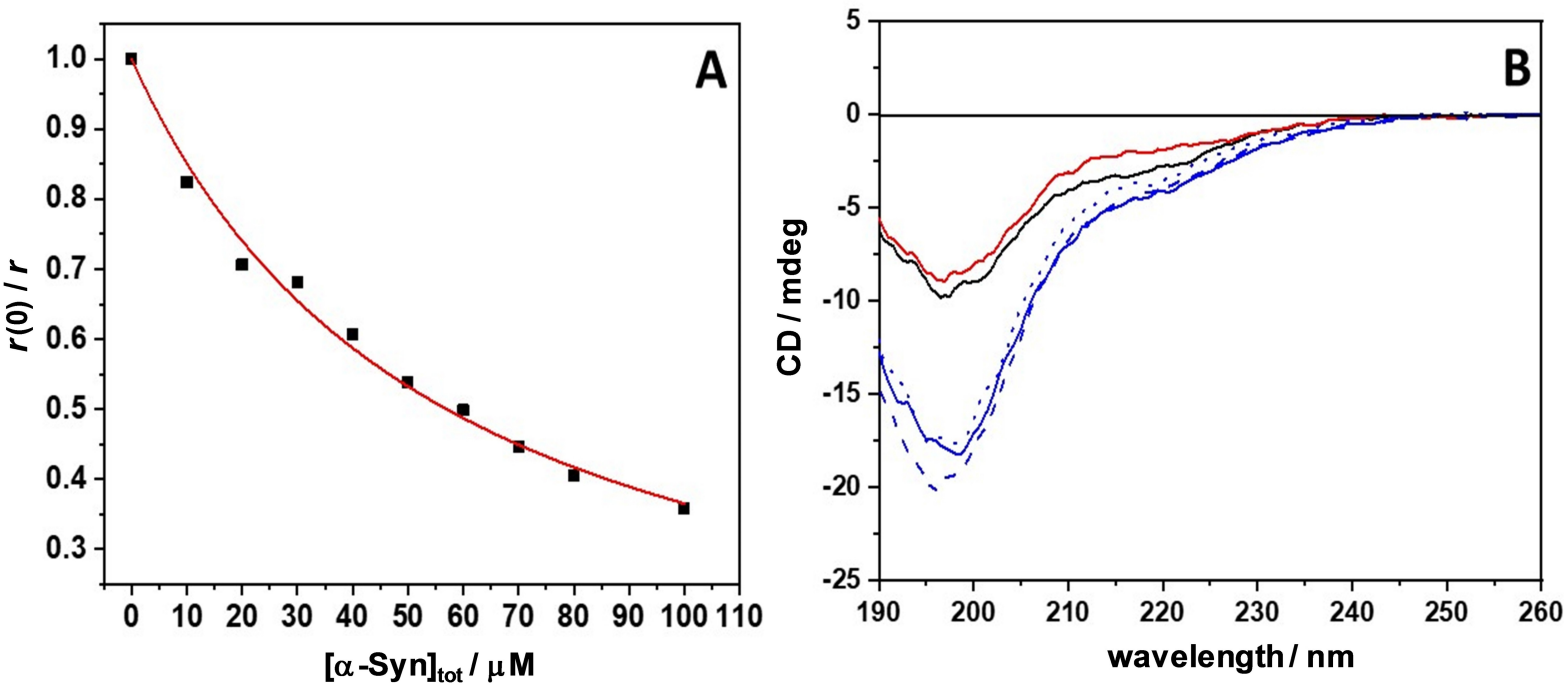
A) Binding isotherm obtained by means of steady‐state fluorescence anisotropy measurements. The experiment was performed by titrating a 4 μM solution of carboxyfluorescein‐labeled LL‐III with a solution of α‐Syn in the range 0–100 μM. The red line represents the best fit according to a 1 : 1 binding model.^[29]^ The fluorophore was covalently attached to the N‐terminus of the peptide. B) Circular dichroism (CD) spectra of LL‐III (black solid line), α‐Syn (red solid line,) and of a mixture of LL‐III and α‐Syn (blue solid line) recorded at day 0. Shown are also spectra of the LL‐III/α‐Syn mixture recorded after 5 days (dashed blue line) and after 10 days (dotted blue lines). Here, the concentration of LL‐III was 300 μM, and the concentration of α‐Syn was 30 μM. All experiments were performed in 20 mM sodium acetate buffer, pH 5.0, at the temperature of 25 °C.

From the data reported so far, it is clear that the LL‐III peptide partitions inside the droplet phase of α‐Syn. To reveal if the peptide is simply partitioning into the droplet phase or if it interacts efficiently with monomeric α‐Syn, we measured the binding affinity of LL‐III to α‐Syn by means of steady‐state fluorescence anisotropy. A 4 μM solution of carboxyfluorescein‐labeled LL‐III was titrated with a solution of monomeric α‐Syn. The fluorescence anisotropy of the labeled peptide was recorded by exciting the samples at 480 nm and evaluating the fluorescence anisotropy at 528 nm, which is the emission maximum of the free peptide. Figure 2A depicts a plot of *r*(0)/*r* as a function of total α‐Syn concentration, where *r*(0) and *r* are the anisotropies of the peptide in the absence and in the presence of α‐Syn, respectively. The data could be well fitted with a 1 : 1 binding model,^[29]^ and the binding constant, *K*_b_, obtained was (1.9±0.3) ⋅ 10^4^ M^−1^. To exclude a possible influence of the extrinsic fluorophore on the interaction process, we performed a complementary titration experiment by recording the anisotropy changes of the Trp residue of LL‐III (Figure S4). The *K*_b_‐value obtained was (3.7±1.0) ⋅ 10^4^ M^−1^, in good agreement with the data obtained from the carboxyfluorescein‐labeled peptide. These data demonstrate that the LL‐III is able to weakly interact with the monomeric form of α‐Syn, leading to the observed growth of the droplets and preventing transformation to the fibrillar state of α‐Syn.

From the magnitude of *K*_b_, the fraction of complexed α‐Syn in the microscopy experiments could be calculated (Figure S5). For 100 μM α‐Syn, the fraction of complexed α‐Syn, *θ*, in the presence of 50 μM LL‐III amounts to about 0.3. At this LL‐III concentration, the formation of fibrils was already inhibited. This is likely due to the ability of the peptide to interact with one or two α‐Syn molecules within the droplets. The interaction is expected to occur mainly via the interaction of the positively charged (+6) and hydrophobic groups of LL‐III with the negatively charged C‐terminus (−12) and hydrophobic NAC region of α‐Syn, respectively. In fact, NMR data showed that cationic polyamines are able to bind to the C‐terminus region of α‐Syn through electrostatic interactions and that the strength of binding depends on the net charge of the polyamines, supporting our conclusions.^[30]^ In the presence of 200 μM and 500 μM LL‐III, the *θ*‐values are 0.70 and 0.90, respectively. At these *θ‐*values, the number of droplets was significantly increased, and droplet growth (via Ostwald ripening or droplet fusion) and their association was promoted.

Next, by means of circular dichroism (CD) spectroscopy, we explored the effect of LLPS formation and LL‐III partitioning on the conformation of the protein. Figure 2B shows the CD spectra of LL‐III, monomeric α‐Syn, and the mixture of LL‐III and α‐Syn. It is important to note that even if the total concentrations of LL‐III and protein are different, their concentrations in terms of amide bonds are similar (∼3.9 mM). The amide bond chromophore is responsible for the observed CD signal, which is able to report on conformational changes of the interacting partners upon binding. An inspection of Figure 2B reveals that both, the antimicrobial peptide and α‐Syn adopt a random‐coiled conformation in solution as evidenced by a minimum of the CD signal around 195 nm, in agreement with previous observations.[^8^, ^31^] When both LL‐III and α‐Syn are present in solution, no conformational changes were observed. In support of this conclusion, we note that the spectrum of the mixture is superimposable with the sum of the spectra of LL‐III and α‐Syn, which were recorded separately (Figure S6). The time evolution of the CD spectrum of the LL‐III/α‐Syn mixture was also recorded after 5 and 10 days (Figure 2B). Even after 10 days, the CD‐spectrum of the LL‐III/α‐Syn mixture showed only features of a random conformation, i. e., at a time point where droplets had formed. This indicates that no significant conformational changes occurred upon formation of the droplet phase, also highlighting the stability of the co‐condensate formed by α‐Syn and LL‐III.

**Figure 3:**
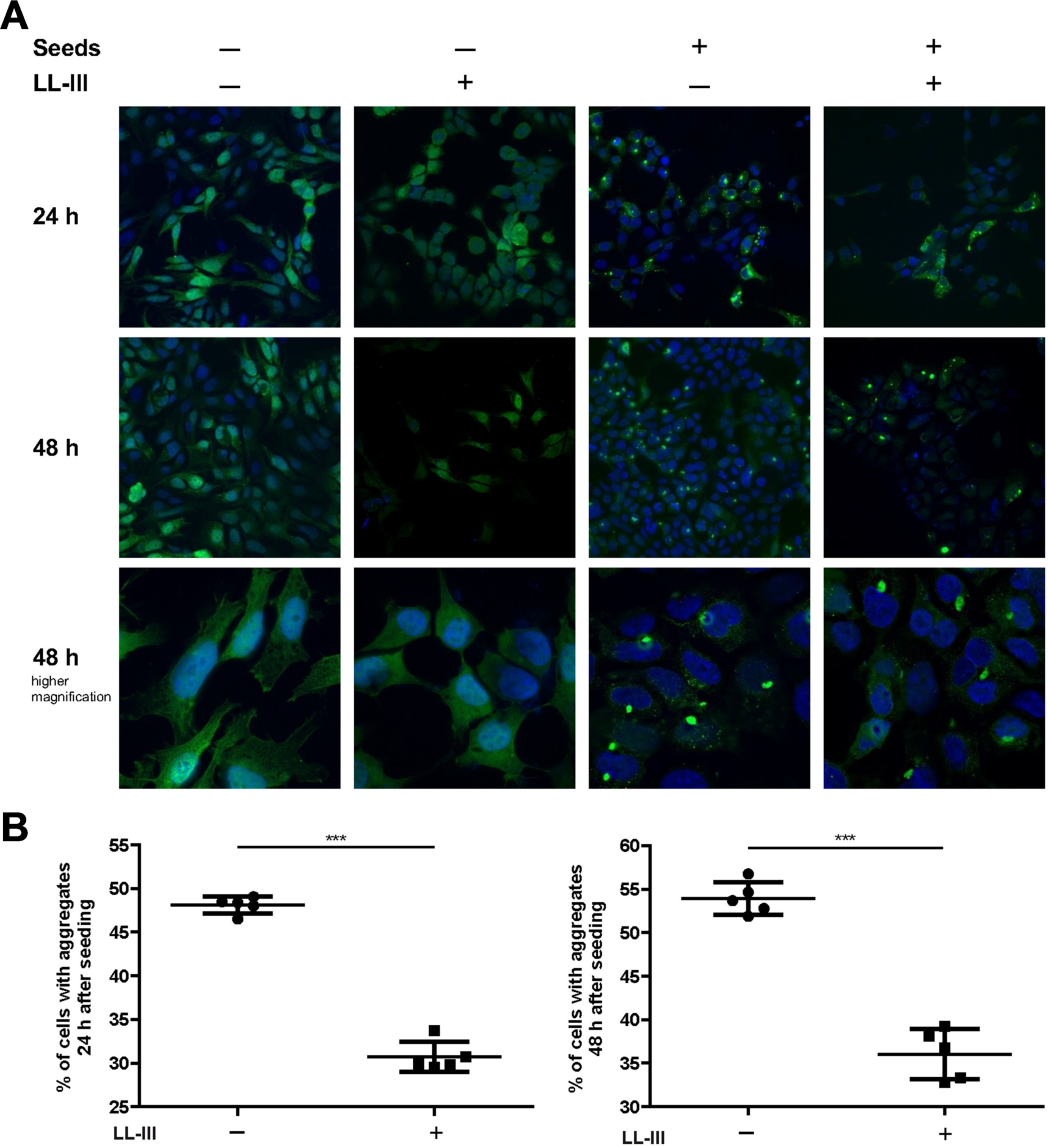
LL‐III decreases PFF‐induced α‐Syn aggregation in neuronal cells. SH‐SY5Y cells stably expressing α‐Syn‐GFP were pre‐treated without or with LL‐III 1 h before adding PFF seeds to induce α‐Syn aggregation. A. α‐Syn was visualized by structured‐illumination microscopy 24 h and 48 h after PFF treatment. Green: α‐Syn (GFP), blue: nuclei (DAPI). B. Quantification of cells with α‐Syn aggregates was based on 5 independent experiments. In total, at least 150 cells were counted per condition. Data were processed by a Kolmogorov‐Smirnov test for Gaussian distribution followed by an unpaired two‐tailed Student's t‐test. *** *p*≤0.001.

Finally, to test the anti‐aggregation activity of LL‐III in a cellular model, we used the α‐Syn pre‐formed fibril (PFF) model, which involves conversion of recombinant monomeric α‐Syn protein into fibrillar seeds that were added to neuronal SH‐SY5Y cells stably expressing an α‐Syn‐GFP fusion protein.[^32^, ^33^] In brief, untreated cells expressed soluble α‐Syn‐GFP that was rather homogenously distributed throughout the cells (Figure 3, left panel). Treatment of cells with PFF seeds caused aggregation of α‐Syn‐GFP, reflected by the formation of GFP‐positive foci (Figure 3, middle and right panel). These α‐Syn‐GFP aggregates could be visualized already 24 h after PFF treatment by fluorescence super‐resolution structured illumination microscopy. Notably, the α‐Syn‐GFP aggregates induced by PFF were phosphorylated at serine 129 which is a pathological signature of α‐Syn aggregates in human synucleinopathies.^[34]^

LL‐III was pre‐incubated with α‐Syn‐GFP‐expressing SH‐SY5Y cells at a concentration of 1 μM one hour before PFF treatment to allow cellular uptake of LL‐III. The number of cells with α‐Syn‐GFP aggregates was quantified 24 h and 48 h after PFF seeding. In both conditions, the number of cells with aggregates was significantly decreased in cells pre‐treated with LL‐III (from 48 % to 30 % after 24 h and from 54 % to 36 % after 48 h). To better recognize the nuclear stain of cells pre‐treated with LL‐III after 48 h, a microscopy picture with enhanced DAPI intensity is reported in Figure S7.

## Conclusions

To conclude, the conversion of α‐Syn from the functional monomeric to the disease‐related amyloid state can probably proceed through two distinct pathways: α‐Syn can initially form small oligomers that convert into larger oligomers, which then grow into amyloid fibrils. In the condensation pathway, pathological protein aggregation of α‐Syn proceeds via formation of droplets, which then gradually mature into the amyloid state, which is seen in the pathological Lewy bodies. Liquid droplet formation of α‐Syn is mainly mediated by an interplay of electrostatic interactions in the unstructured N‐terminal domain and hydrophobic interactions in the NAC region. During maturation and ordered fibril formation, the C‐terminal domain of α‐Syn becomes also involved.^[8]^ Which route is followed, is likely also determined by the environmental conditions, viz. the ionic strength, pH, osmolytes, metal ion exposure, crowding, and nucleation by the presence of lipid interfaces and filaments.[^10^, ^11^, ^12^, ^13^, ^14^, ^35^]

**Figure 4:**
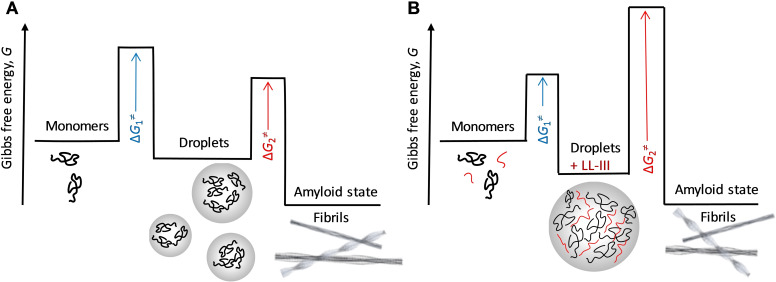
a) Schematic Gibbs free energy diagram of α‐Syn upon maturation from its monomeric to the droplet and finally fibrillar state in the absence (A) and presence (B) of the antimicrobial peptide LL‐III. The amyloid state is most likely the thermodynamically most stable state, but its kinetic accessibility is reduced by the presence of free energy barriers, Δ*G* ^≠^, between the native (monomers) and droplet state, and between the droplet and amyloid (fibrillar) state. The latter free energy barrier, Δ*G*_2_^≠^, can be crossed through a maturation process, eventually involving the formation of gel‐like assemblies that gradually age into the amyloid state. In the presence of the antimicrobial peptide LL‐III, the conversion to the amyloid state can be stopped owing to an increase of the stability of the droplet state and a significant increase of the free energy barrier to the fibrillar state. Here we assumed that the (concentration‐dependent) free energy of the fibrillar amyloid does not change in the presence of LL‐III.

The amyloid state is generally thought to be the thermodynamically most stable state, but its kinetic accessibility is reduced by the presence of free energy barriers between the native and droplet state, Δ*G*_1_^≠^, and between the droplet and amyloid state, Δ*G*_2_^≠^ (see schematic in Figure 4). The latter free energy barrier, Δ*G*_2_^≠^, can be crossed through a maturation process that involves the formation of hydrogel‐like β‐sheet‐rich assemblies which gradually age into the fibrillar amyloid state.

Here, we show to the best of our knowledge for the first time, how LLPS and droplet formation of α‐Syn is affected by an antimicrobial peptide. We show that the antimicrobial peptide LL‐III is able to interact efficiently with liquid phase droplets of α‐Syn. Serving as weak cross‐linkers between α‐Syn molecules, LL‐III stabilizes the droplet condensate and prevents transformation to the fibrillar amyloid state by increasing the droplet stability and increasing the activation free energy barrier leading to the fibrillar state. No decondensation of the droplets takes place. The anti‐aggregation activity of LL‐III was also observed in a cellular model. We anticipate that the study of antimicrobial‐like peptides interacting with specific liquid condensates such as that of α‐Syn will generally contribute to the understanding of complex disease mechanisms emanating from such condensates, and may also open up exciting new avenues for intervention.

## Conflict of interest

The authors declare no conflict of interest.

## Supporting information

As a service to our authors and readers, this journal provides supporting information supplied by the authors. Such materials are peer reviewed and may be re‐organized for online delivery, but are not copy‐edited or typeset. Technical support issues arising from supporting information (other than missing files) should be addressed to the authors.

Supporting Information
