# Supplementary material for: Remodeling of the Fibrillation Pathway of α‐Synuclein by Interaction with Antimicrobial Peptide LL‐III
Source: Chemistry. 2021 Jul 22;27(46):11845–51. doi: 10.1002/chem.202101592 (PMC8457056; doi:10.1002/chem.202101592)
Supplement: Supplementary file 1 — Supporting Information [file CHEM-27-11845-s001.pdf]

Chemistry–A European Journal

Supporting Information

Remodeling of the Fibrillation Pathway of α-Synuclein by Interaction with Antimicrobial Peptide LL-III

Rosario Oliva,* Sanjib K. Mukherjee, Lena Ostermeier, Lilli A. Pazurek, Simon Kriegler, Verian Bader, Daniel Prumbaum, Stefan Raunser, Konstanze F. Winklhofer, Jörg Tatzelt, and Roland Winter*

Materials and Methods

Materials

The lyophilized powders of the labeled and unlabeled LL-III peptide were purchased from GenScript, Germany. The sequence of the LL-III peptide is NH_2-VNWKKILGKIIKVVK-$CONH_2$. In the labeled peptide, the fluorophore 6-carboxyfluorescein (FAM) was covalently attached to the N-terminus of the peptide. All the solutions were prepared in 20 mM sodium acetate buffer at pH 5.0. Deionized water was used for the buffer and all sample preparations.

Expression and purification of α-synuclein

The expression and purification of α-synuclein (α-Syn) was carried out as described before.[1,2] The plasmid was purchased from GenScript. The plasmid pT7-7 expressing human α-synuclein was transformed into *Escherichia coli* strain BL21 (DE3). A single colony was picked and inoculated into 100 ml LB medium containing 150 µg/ml ampicillin and grown at 37°C with shaking at 250 rpm until the absorbance at 600 nm reached 0.8. Induction was then carried out by adding 1 mM IPTG (final concentration) and the culture was further grown under similar conditions for 3 h. The cells were harvested, resuspended in 0.75 ml of buffer (50 mM Tris-HCl, pH 7.5, 10 mM EDTA and 150 mM NaCl) and frozen at -80 °C. Tubes containing frozen cells were placed in a boiling water bath for 7 min and the supernatant collected after centrifugation at maximum speed for min. Streptomycin sulfate (136 µl/ml of supernatant) and glacial acetic acid (228 µl/ml of supernatant) were added and centrifuged for 2 min. Again, the supernatant was recovered and precipitated with ammonium sulfate (saturated ammonium sulfate at 4°C was used 1:1, v/v, with supernatant). The protein was collected as a precipitate by centrifugation and washed once with 1 ml of ammonium sulfate solution (4°C, 1:1, v/v, saturated ammonium sulfate and water). The washed pellet was resuspended in 900 µl of 100 mM ammonium acetate (to form a cloudy solution) and precipitated by adding an equal volume of ethanol at room temperature. Precipitation with ethanol was repeated once more. The pellet was resuspended in 100 mM ammonium acetate and extensively dialyzed against 10 mM Tris-HCl buffer, pH 7.4.

Phase-contrast light and fluorescence microscopy

The light and fluorescence microscopy experiments were performed using an Eclipse TE2000-U (Nikon Inc.) optical microscope with a Nikon Plan Fluor 20x objective (NA 0.45 WD 7.4) coupled to an TIS DMK 23UX249 camera. For the microscopy experiments, a cell with flat diamond windows as optical window material on both sides was used at a temperature of 25 °C, which was controlled by a circulating water bath. The concentration of α-synuclein was 100 µM. Samples in the absence and in the presence of LL-III at the concentration of 50, 200 and 500 µM were prepared. Fluorescence microscopy experiments were performed using 6-carboxyfluorescein-labeled LL-III (FAM-LL-III) exciting at 488 nm. In these experiments, 1 µM solution of FAM-LL-III was mixed with the unlabeled peptide to reach final peptide concentrations of 50, 200, and 500 µM.

Atomic Force Microscopy (AFM)

AFM measurements were carried out on a MultiMode scanning probe microscope with a Nano-Scope IIIa controller (Digital Instruments, Santa Barbara, CA) and a J-Scanner (maximum scan size 125 µm). Images were obtained using the tapping-mode in 20 mM sodium acetate buffer at pH of 5.0, with silicon SPM sensor/Nanosensors. Several µL of 20 µM α-Syn solution in the absence and in the presence of 200 and 500 µM LL-III were dried on a mica surface before taking the images. The quality of fibril images could possibly be affected by the drying process on the mica surface of the AFM stage. Tips with nominal force constants of 10-130 Nm^{-1} were used at driving frequencies of 200-500 kHz and drive amplitudes between 50 and 500 mV. The scan frequency was 1.49 Hz. Images were taken with a resolution of 512 × 512 pixels. All AFM experiments were carried out at room temperature and analyzed using NanoScope version 5.

Steady-state fluorescence anisotropy

Fluorescence anisotropy experiments were performed by means of a K2 fluorometer from ISS (Champaing, IL, USA) using a quartz cuvette with a path length of 0.3 cm and a final volume of 60 µL. The temperature was set at 25 °C by using a water circulating bath connected directly to the sample compartment. For the determination of the binding affinity between LL-III and LAF-1, steady-state fluorescence anisotropy data of a 4 µM solution of LL-III labeled with carboxyfluorescein were recorded, varying the α-synuclein concentration in the range 0-100 µM. The excitation and emission wavelengths were set at 480 nm and 528 nm, respectively. Each anisotropy value is the average of at least 10 acquisitions. As control, the same experiment was repeated by using the unlabeled peptide (5

μM) and following the anisotropy changes of the Trp residue. The α-synuclein concentration was varied between 0 and 50 μM. The excitation wavelength was set to 295 nm in order to avoid excitation of the Tyr residues of the α-synuclein (note that α-synuclein has no Trp in its sequence). The anisotropy was evaluated at 354 nm which corresponds to the maximum of emission of the free peptide. Then, the binding isotherms were obtained by plotting $r(0)/r$ as a function of total α-synuclein concentration. Here, $r(0)$ and r denote the anisotropies of the peptide in the absence and in the presence of a given α-synuclein concentration, respectively. In order to evaluate the value of the binding constant, K_b, the experimental data were fitted by using a 1:1 binding model.

Circular dichroism spectroscopy

Far-UV circular dichroism (CD) spectra were recorded by using a Jasco J-715 spectropolarimeter from Jasco Corporation (Tokyo, Japan). All the spectra were acquired at the temperature of 25 °C using a 0.01 cm path length quartz cuvette. The following parameters were used for spectra acquisition: scan rate of 50 nm min^{-1}, response time of 2 s, and a band width of 5 nm. Spectra of α-synuclein (concentration of 30 μM), LL-III (300 μM) and α-Syn+LL-III at the same concentrations were acquired. A blank spectrum of 20 mM sodium acetate buffer, pH 5.0, was recorded and subtracted from all the samples spectra. The reported spectra are the results of 4 accumulations.

Preparation of negative stain specimens and electron microscopy

Negative stain specimens were prepared as described previously.[3] A 5 μL volume of the α-Syn sample were absorbed at 25 ºC for 5 min onto a freshly glow-discharged 400 mesh carbon-coated copper grids (G2400C, Plano GmbH, Wetzlar, Germany). After the pre-incubation time, excess sample was blotted by touching a Whatman filter paper and washed with three droplets of ddH_2O and exposed to freshly prepared 1% uranyl acetate solution (SERVA Electrophoresis GmbH, Heidelberg, Germany) for about 1 min. Excess negative stain solution was blotted and the specimen air-dried.

Specimens were inspected with a JEM1400 microscope (Jeol, Tokio, Japan) equipped with a LaB_6 cathode and operated at an acceleration voltage of 120 kV. Digital micrographs were recorded at pixel sizes of 2.8 Å/pix and 5.6 Å/pix with a 4k x 4k CMOS camera F416 (TVIPS, Gauting, Germany).

Cell culture and seeding

The generation of the stable cell line expressing EGFP-α-SynA53T and seeding was described previously.[4] In brief, for the generation of seeding-competent α-Syn assemblies, purified α-Syn monomers (5 mg/mL) were centrifuged at high speed (100,000 × *g*) for 1 h. The supernatant was transferred into a new reaction tube, incubated with constant agitation (900 r.p.m.) at 37 °C for 24 h. The resulting assemblies were diluted 1:20 in Opti-MEM (ThermoFischer) and sonicated for 3 min using a Branson sonifier. For seeding, the sonicated α-Syn assemblies were diluted into a mixture of 1 Opti-MEM and 40 μl of Lipofectamine 2000 (ThermoFischer). After incubation for 15 min, 200 μL of the suspension was added to cells cultivated in 1800 μL Opti-MEM medium.

Super-Resolution Structured Illumination Microscopy (SR-SIM)

Cells were fixed with 4% ice-cold paraformaldehyde (PFA) after 24 h or 48 h of seeding and mounted on coverslips using a solution of DAPI and Fluoromount-G™ Mounting medium. Fluorescence microscopy was performed using a ZEISS ELYRA PS.1 microscope equipped with an LSM880. For SR-SIM image acquisition, a 20x and a 63x oil immersion objective were used to acquire raw images. ZEN Black 2.1 SP3 was employed to generate super-resolution images.

[1] M. J. Volles, P. T. Lansbury, *J. Mol. Biol.* **2007**, *366*, 1510-1522.

[2] R. Shaltiel-Karyo, M. Frenkel-Pinter, N. Egoz-Matia, A. Frydman-Marom, D. E. Shalev, D. Segal, E. Gazit, *PLoS One* **2010**, *5*, e13863.

[3] C. Bröcker, A. Kuhlee, C. Gatsogiannis, H. J. kleine Balderhaar, C. Hönscher, S. Engelbrecht-Vandré, C. Ungermann, S. Raunser, *Proc. Natl. Acad. Sci. USA* **2012**, 109, 1991-1996.

[4] V. A. Trinkaus, I. Riera-Tur, A. Martinez-Sanchez, F. J. B. Bauerlein, Q. Guo, T. Arzberger, W. Baumeister, I. Dudanova, M. S. Hipp, F. U. Hartl, R. Fernandez-Busnadiego, *Nat. Commun.* **2021***, 12*, 2110.

Supporting Figures

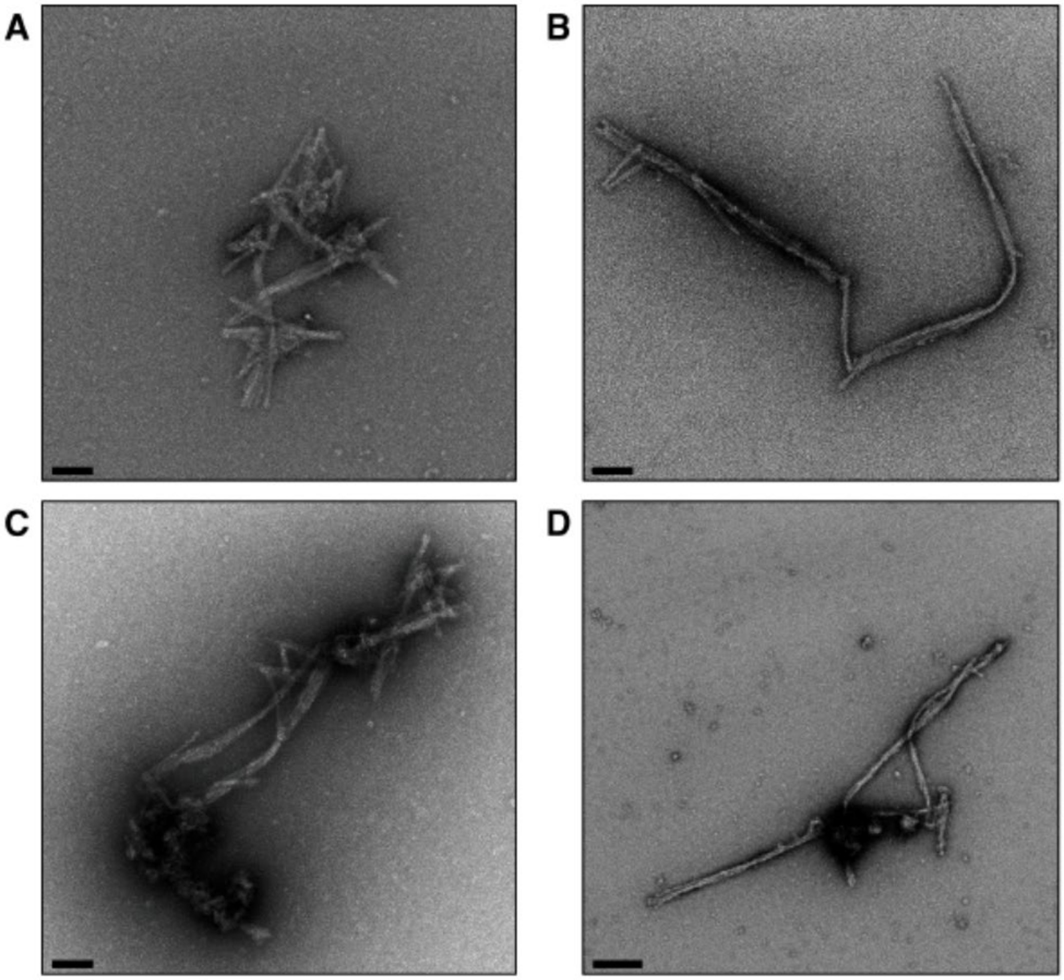

Figure S1. Transmission electron microscopy (TEM) micrographs showing fibers of α-Syn formed after 9 days of incubation of the protein in its monomeric state. In panel (A), (B) and (C), the scale bar is 100 nm. Instead, in panel (D) the scale is 200 nm. For sample preparation and image acquisition, please refers to the Materials and Methods section.

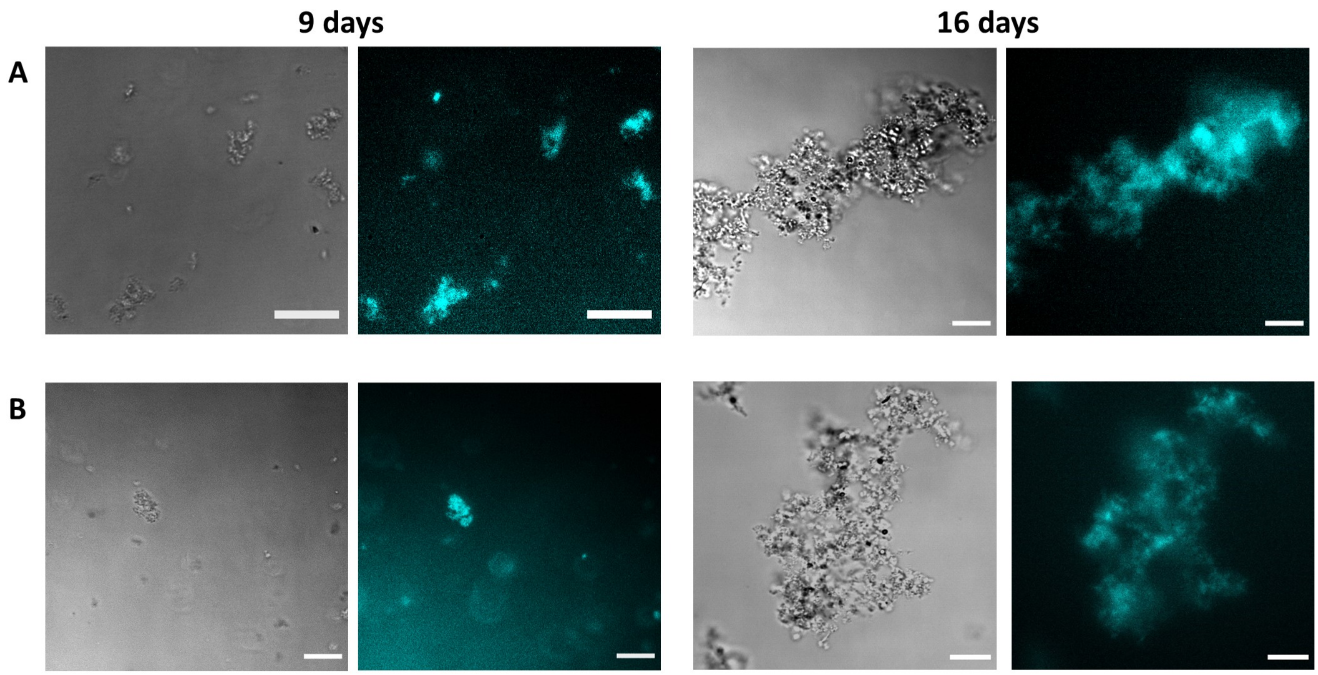

Figure S2. Phase-contrast light and fluorescence microscopy snapshots of a 100 µM α-Syn solution in the presence of (A) 200 µM and (B) 500 µM LL-III (total peptide concentration, the concentration of the carboxyfluorescein-labelled peptide was 1 µM). All the experiments were performed at room temperature (25 °C) in 20 mM sodium acetate buffer, pH 5.0. The scale bar is 30 µm.

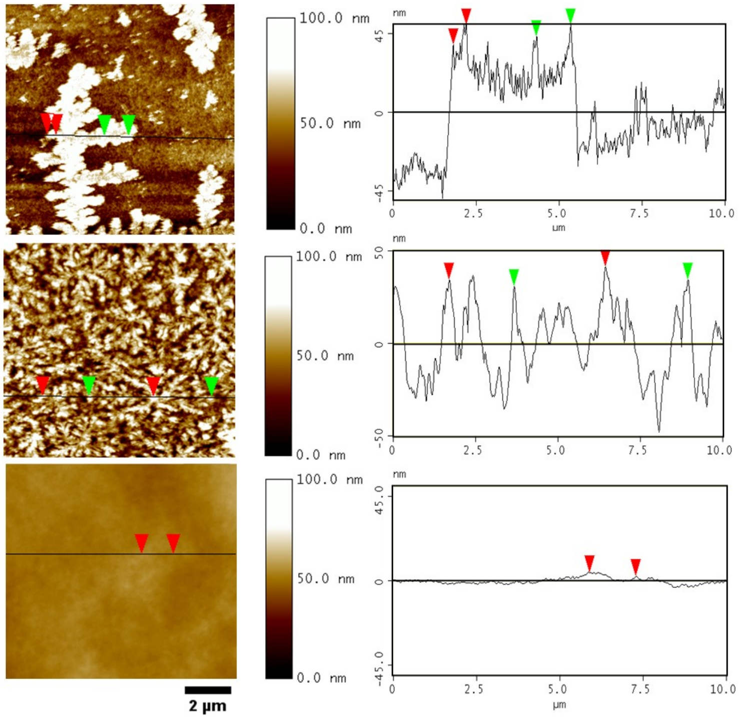

Figure S3. AFM images and height profiles of dried α-Syn solution in the absence (top panel) and in the presence of 200 µM (middle panel) and 500 µM (bottom panel) LL-III. The thick fibrils are composed of several thinner fibrils associated laterally.

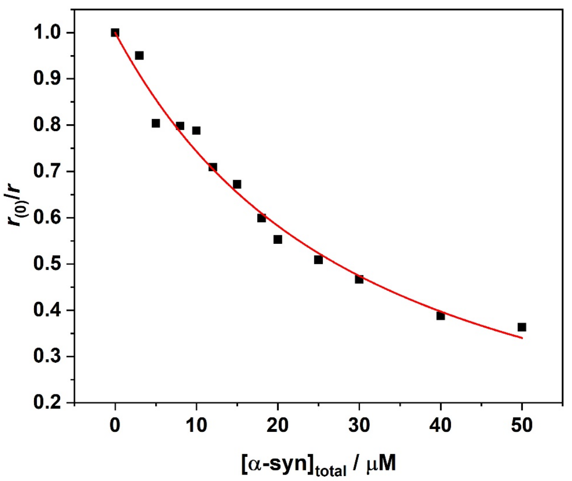

Figure S4. Binding isotherm obtained by means of Trp steady-state fluorescence anisotropy. The experiment was performed by titrating a 5 μM solution of LL-III with a solution of α-Syn in the range 0-50 μM. The red line represents the best fit according to a 1:1 binding model. The experiments were performed in 20 mM sodium acetate buffer, pH 5.0, at 25 °C.

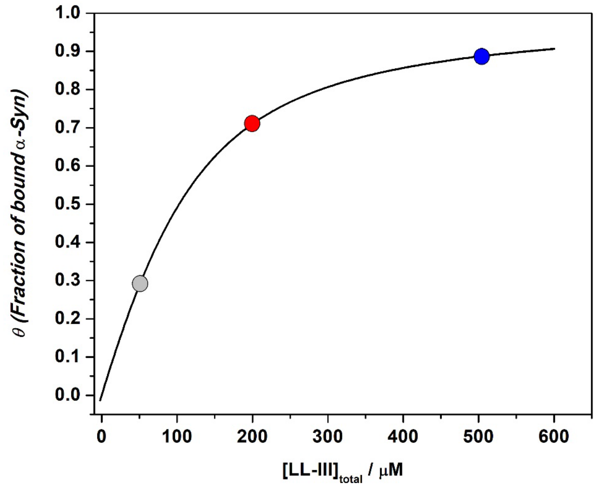

Figure S5. Plot of the fraction of bound α-Syn as a function of the total peptide concentration. The fraction of bound α-Syn was calculated according to the concentration used in the phase-contrast light microscopy experiments reported in Figure 1B of the main text: 100 μM α-Syn and 50 μM (grey circle), 200 μM (red circle) and 500 μM (blue circle) of LL-III.

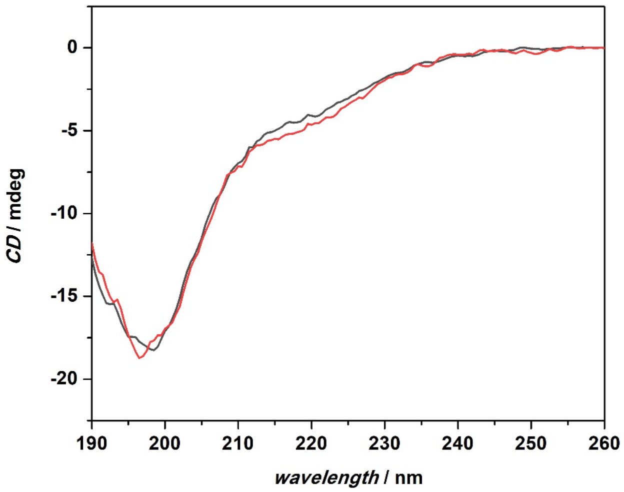

Figure S6. Superimposition of the CD spectra of the α-Syn/LL-III mixture (black line) and of the spectrum obtained by summing the CD spectra of LL-III and α-Syn (red line) recorded separately (black and red spectra reported in Figure 2B).

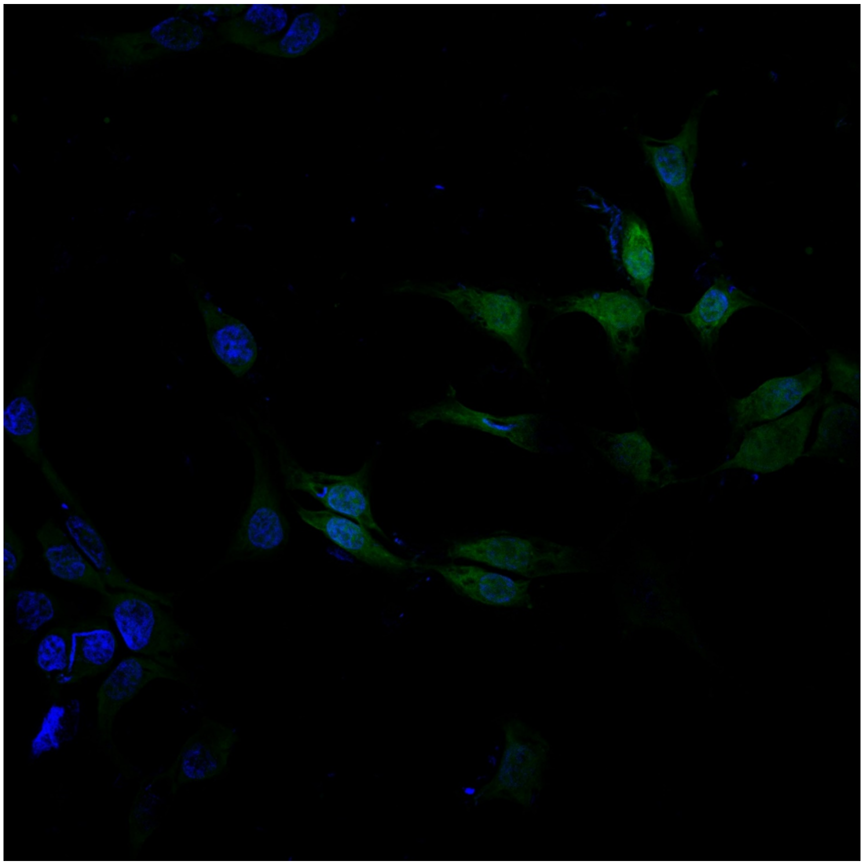

Figure S7. Structured-illumination microscopy picture of SH-SY5Y cells pre-treated with LL-III peptide. The picture was taken after 48 h from peptide addition. In this picture, to better recognize the nuclear staining, the DAPI intensity was enhanced.
